# A Game Theoretic Approach for Balancing Energy Consumption in Clustered Wireless Sensor Networks

**DOI:** 10.3390/s17112654

**Published:** 2017-11-17

**Authors:** Liu Yang, Yinzhi Lu, Lian Xiong, Yang Tao, Yuanchang Zhong

**Affiliations:** 1 School of Communication and Information Engineering, Chongqing University of Posts and Telecommunications, Chongqing 400065, China; xionglian@cqupt.edu.cn; 2 School of Electronic Information Engineering, Yangtze Normal University, Chongqing 408100, China; henanluyinzhi@163.com; 3 College of Communication Engineering, Chongqing University, Chongqing 400044, China; zyc@cqu.edu.cn

**Keywords:** wireless sensor networks (WSNs), clustering, network lifetime, game theory, equilibrium

## Abstract

Clustering is an effective topology control method in wireless sensor networks (WSNs), since it can enhance the network lifetime and scalability. To prolong the network lifetime in clustered WSNs, an efficient cluster head (CH) optimization policy is essential to distribute the energy among sensor nodes. Recently, game theory has been introduced to model clustering. Each sensor node is considered as a rational and selfish player which will play a clustering game with an equilibrium strategy. Then it decides whether to act as the CH according to this strategy for a tradeoff between providing required services and energy conservation. However, how to get the equilibrium strategy while maximizing the payoff of sensor nodes has rarely been addressed to date. In this paper, we present a game theoretic approach for balancing energy consumption in clustered WSNs. With our novel payoff function, realistic sensor behaviors can be captured well. The energy heterogeneity of nodes is considered by incorporating a penalty mechanism in the payoff function, so the nodes with more energy will compete for CHs more actively. We have obtained the Nash equilibrium (NE) strategy of the clustering game through convex optimization. Specifically, each sensor node can achieve its own maximal payoff when it makes the decision according to this strategy. Through plenty of simulations, our proposed game theoretic clustering is proved to have a good energy balancing performance and consequently the network lifetime is greatly enhanced.

## 1. Introduction

A conventional wireless sensor network (WSN) [1] is composed of a mass of tiny, cheap and low-powered sensor nodes with limited sensing, processing and transmitting abilities. After random deployment in a sensing area, these sensor nodes can construct a self-organizing network system automatically through wireless communication [2]. WSNs have been applied in various domains, such as natural disaster relief [3], environmental monitoring [4], biomedical health monitoring [5], military target tracking [6] and smart homes [7], aiming to gather information from the physical environment of interest. WSNs usually are distributed in harsh or hostile environments and work in an unattended way [8]. Sensor nodes in the network are usually powered by batteries with limited capacity, it is hard to recharge or replace the battery after the energy is exhausted. Energy constraints are an essential issue that severely restricts the network lifetime [9]. Since information transmission and receipt exhausts the majority of the energy of sensor nodes [10], an energy-efficient routing scheme need to be adopted to effectively reduce the energy consumption. When designing routing protocols, the major objectives are minimizing energy dissipation of sensor nodes and maximizing the network lifetime [11]. As a more effective way to optimize the network lifetime than other routing schemes, clustering has attracted a lot of attention in the past decade [12,13,14]. The idea of clustering is that sensor nodes are clustered into several groups in the network, and each cluster contains one cluster head (CH) node and some normal cluster member (CM) nodes [15]. CMs only transmit the data to their own CHs which have the responsibilities of receiving, aggregating and forwarding these data. Then the communication cost is greatly reduced since only the aggregated data packets need to be forwarded to the base station (BS) over a long distance by CHs. However, the network lifetime can be greatly weakened if a sensor node acts as the CH frequently, since CHs always consume much more energy than normal nodes. Thus, energy consumption must be balanced among sensor nodes to enhance the network lifetime for clustered WSNs.

Game theory [16,17] is a powerful mathematical tool used to analyze and predict the decisions of intelligent rational individuals under conflict situations. It has been widely applied in economics, politics and sociology. Recently, it has been introduced to solve the clustering problem in WSNs by designing energy-balanced CHs optimization mechanisms [18,19,20]. Each node is modeled as a rational decision-maker in the network, which has the ability to make decisions based on its own interests. On the one hand, each node abdicates the duty of serving as a CH to other nodes for energy conservation purposes, since a CH has higher energy consumption rate than a normal sensor node; On the other hand, at least one sensor node has to be selected as the CH to provide services for other nodes within a local area. Due to this conflict, game theory is very suitable to make decisions for sensor nodes. Mixed strategies are usually adopted by sensor nodes in game theoretic clustering, such as CROSS [19] and LGCA [20], where each sensor node decides whether being a CH based on a probability. Game theory is used to find an equilibrium solution among the mixed strategies. By playing the clustering game, each sensor node acquires an equilibrium probability to be the CH. And it decides whether to act as the CH according to this probability so that it has no incentives to deviate from the selected strategy as a tradeoff between providing services and saving energy is achieved. However, how to get the equilibrium strategy while maximizing the payoff of each node is rarely addressed to date. In this paper, game theory will be used to model the clustering for WSNs. And we devote to searching for the Nash equilibrium (NE) solution with regards to the combination strategies while maximizing the payoff of each sensor node. Here we list our main contributions as follows:

- We present a game theoretic approach for balancing energy consumption in clustered WSNs. Where sensor nodes are modeled as rational and selfish players and the ones in a close neighbor will participate in a clustering game for CH election;
- In our clustering game, we construct a novel convex payoff function for each sensor node that can capture the node’s realistic behaviors. Through convex optimization, the equilibrium solution of this game is derived. Each node makes its own decisions based on the result of this equilibrium solution to achieve a tradeoff between providing data forwarding services and saving energy, and meanwhile, its own payoff can be maximized;
- Considering the energy heterogeneity of sensor nodes in practical scenario, we introduce a penalty mechanism to compel the sensor nodes which hold more energy to compete for the CHs more actively;
- Through extensive simulations under various conditions, we prove that the performance of our protocol outperforms the recent game theoretic clustering protocols CROSS and LGCA.

We organize the rest of this paper as follows: Section 2 summarizes the works most related to our clustering protocol. Section 3 discusses the system model. A CH election game is presented in Section 4. The details of our clustering protocol are described in Section 5. We give the performance evaluation of our protocol in Section 6. Finally, conclusions and further work are shown in Section 7.

## 2. Related Works

Clustering techniques for WSNs have been widely studied recently for the goal of prolonging the network lifetime. In this section, we will provide a review of some clustering schemes most related to our work.

Low Energy Adaptive Clustering Hierarchy (LEACH), one of the most famous traditional clustering protocols, was presented in [21]. All sensor nodes in the network are clustered into some groups. A CH is selected in each cluster with the responsibilities of receiving, aggregating and forwarding data from other normal nodes distributed in the same cluster. Since CHs have more data traffic and consume much more energy than normal sensor nodes, the CH role must be rotated among nodes to balance the energy. Then a random CH rotation mechanism is introduced so that a sensor node can serve as the CH once within a specific time interval. This mechanism can balance the energy dissipation among sensor nodes, but it may result in an uneven distribution of clusters. Another famous clustering protocol called Hybrid Energy-Efficient Distributed clustering (HEED) was presented in [22]. CHs are periodically selected by adopting an iterative CH rotation mechanism. The residual energy of nodes and communication cost within a cluster are considered when selecting the suitable CHs. Then sensor nodes which possess more energy have bigger probabilities to be CHs, and each one prefers to join the cluster with smaller communication cost. Furthermore, this CH rotation mechanism can assure that no more than two sensor nodes within a close neighborhood will be selected as the CH simultaneously. Finally, HEED acquires a more even distribution of clusters than LEACH, but it needs extra energy for the iterative CH election process.

Inspired by behavior of honeybee swarms, a cluster-based routing algorithm for WSNs named queen-bee evolution algorithm (QEGA) was presented in [23]. The queen-bee algorithm has a high execution rate that results in premature convergence. Two mutation rates are introduced to solve this problem, which can increase the diversity of children. QEGA has enhanced energy efficiency and network lifetime compared with genetic algorithm-based clustering, but it does not consider sensor nodes’ residual energy, which is the most important factor in routing protocols for WSNs. A novel ring-based clustering method called Efficient Cluster-Based Communication Protocol (ECOMP) was proposed in [24]. Each cluster consists of some normal sensor nodes, a CH node and a cluster sender node. A bidirectional ring is constructed by normal nodes and the cluster sender node within each cluster. The cluster sender node starts data collection from the normal sensor nodes in a clockwise or anticlockwise direction around the ring in turn. Each normal sensor node aggregates its previous neighbor node’s data with its own, and then forwards the data to its next neighbor. Finally, the cluster sender node has received the data of all normal sensor nodes on the ring, and it transmits the data to a BS by single or multi-hop forwarding with the CHs in the network. ECOMP liberates the CHs from heavy data receiving and aggregating for normal sensor nodes, as each normal node only transmits the data to its near neighbor. Thus the energy dissipation of nodes can be effectively reduced. However, extra costs have to be paid for ring construction, since sensor nodes exchange some information with BS and get locations by GPS. To prolong the stable period of Fog-supported WSNs, a modified Stable Election Protocol (SEP) which is called Prolong-SEP (P-SEP) was proposed in [25]. The whole sensor field is covered by some fog nodes which have the responsibility of forwarding data to the internet gateway. Sensor nodes with two-level heterogeneity about initial energy are pre-placed or randomly deployed into each fog node covered area, and they can be classified into advanced nodes and normal ones. For energy saving purposes, a cluster-based topology is constructed in each covered area, and CHs only need to transmit the aggregated data to the corresponding fog node. To select the appropriate CHs from the network, two energy thresholds corresponding to advanced and normal nodes are designed that both initial energy and current average energy of sensor nodes are considered. Then sensor nodes with higher energy level become CHs more frequently if they have more residual energy than the corresponding threshold. Moreover, information about sensor-to-sensor link gains and inter-sensor distances is exploited to find a proper data transmission path from sensor nodes to the log nodes. Simulation results show that P-SEP has improved the network lifetime by 31% compared with SEP. However, only two-level heterogeneity about initial energy is considered in P-SEP, and pre-deployment of advanced nodes makes it quite limited for execution in practical scenarios. In [26], clustering techniques were used to solve the feedback problem in ultra-wideband systems, where large amounts of feedback are required when transmitting the channel impulse response from receiver to transmitter. By adopting machine learning methods, the estimated channels are clustered into several groups and correspondingly the appropriate channel cluster head (CCH) is selected within each group. Only the label of the most similar CCH to that estimated channel is required for feedback. Thus, the amount of data is reduced significantly across the feedback channel.

As an effective way to make decisions for intelligent rational individuals under conflict situations, game theory has been used to solve the data forwarding problem for sensor nodes in WSNs. A game theory protocol named Forwarding Dilemma Game (FDG) was presented in [27] for the purpose of controlling the overhead during data transmission in multi-hop networks. When a sensor node has received a flooding packet, it decides whether to forward this packet by playing the game with other nodes which have received the same packet. Each player has two strategies: One is forwarding the packet and the other is dropping the packet. The mixed NE strategy is adopted in the game and each player acquires a forwarding probability after this game is finished. Then each player decides whether to forward the packet based on this probability so that its payoff can keep a state of equilibrium. In [28], game theory was used to address the issue of energy balancing in cluster-based WSNs for the intruding detection application. When an event is detected by the nodes within a cluster, it should be reported timely to the sink to activate defensive mechanisms. To balance the energy dissipation among sensor nodes, report tasks in the network need to be fairly assigned. Game theory is used to model the report task assignment process, where sensor nodes in the same cluster are considered as the players for event reporter election. The NE solution is finally derived through convex optimization. Each sensor node decides whether to report the intruding event to BS based on the result of the NE solution, so that its payoff can be maximized while the energy balancing objective is also achieved.

Recently, game theory was introduced to address the clustering for WSNs. A territory game theoretical clustering protocol (TGCP) was presented in [29], where the whole network is divided into a total of four continuous regions A, B, C and D. To balance energy consumption in multi-hop communication networks, sensor nodes in the region near to BS have bigger probability to be CHs than the ones in the region farther from BS. To determine the limit between neighboring regions, an evolution game is introduced to model neighbor regions as two players for territory size contest. TGCP can effectively improve the network performance compared with LEACH, since it alleviates unbalanced energy dissipation due to asymmetrical distances from CHs to BS. However, only four regions are considered in TGCP making it not very suitable for large-scale networks. A protocol called Clustered Routing for Selfish Sensors (CROSS) was presented in [19], where game theory was used to model the clustering for sensor networks. As CHs consume much more energy than normal nodes, a node selfishly abdicates from serving as the CH to other nodes for energy conservation. However, if each node refuses to be the CH, then all sensor nodes’ payoffs are zero since their sensing data cannot be transmitted to BS effectively. To achieve a tradeoff between saving energy and providing the required services, each sensor node is considered as a player to campaign for the CH by joining in a clustering game. The mixed strategy is adopted in this game, and each player acquires an equilibrium probability to be CH after the game is finished. Each node decides whether to be a CH according to the corresponding equilibrium probability so that its payoff can keep the state of equilibrium. Though CROSS has shown a good paradigm to model the clustering in sensor networks by using game theory, the main problem is that it assumes all sensor nodes in the network can exchange information with each other which is not very realistic. To address this problem, a protocol called Localized Game theoretical Clustering Algorithm (LGCA) was presented in [20]. By playing a localized clustering game with the close neighbors, each sensor node can get an equilibrium probability to be the CH and selfishly decides whether to be the CH according to this probability. To avoid the case that more than one CH occur in a localized restricted area, the nodes successfully self-selected as CHs need to further run to become the final CHs. Then a MAC contention mechanism is adopted to insure that only one final CH is restricted in a specific zone. Compared with CROSS, LGCA is more feasible since it is completely distributed and easily extendible. As the payoffs for a sensor node when choosing different strategies are not specifically defined from the view of sensors’ energy in CROSS and LGCA, a Hybrid, Game Theory-based and Distributed (HGTD) clustering protocol was proposed to address this issue in our previous work [30]. Each node plays the same localized clustering game as that in LGCA. Since the nodes with more neighbors and longer distances to BS have higher costs to serve as CHs, node degree and the distance to BS are considered simultaneously when defining nodes’ payoffs. Moreover, an iterative algorithm is presented to restrict that only one CH can be selected in a localized restrict. Extensive simulation results validate that HGTD has further enhanced the network lifetime.

## 3. System Model

In this section, the system model is discussed in detail which contains network model and radio model.

### 3.1. Network Model

In this paper, we study how to use game theory to address the clustering issue in WSNs for energy balancing. To model the network in a real scenario, we assume sensor nodes are heterogeneous in their initial energy in the network. This assumption is very reasonable because that on the one hand, no routing protocols can achieve ideal energy balancing among nodes in the network, and on the other hand, new nodes joining and old nodes leaving also results in energy heterogeneity. We express the initial energy *E_i_* for any sensor node *i* as follows:

(1) $$E_{i}=E_{b}+\alpha \cdot rand$$

where *E_b_* is the basic energy; *α* is the random energy exponent and *rand* is a number randomly selected within the range of (0, 1).

**Figure 1:**
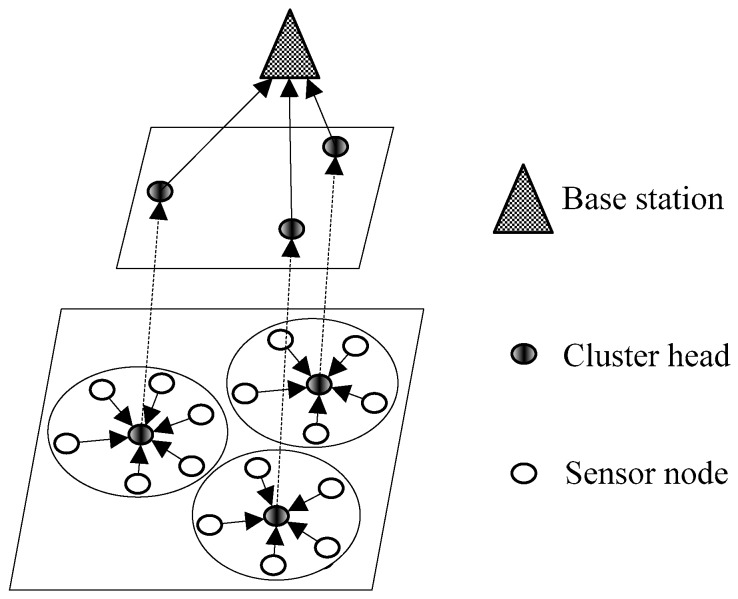
Network model.

We consider the three-level architecture of a model network [30,31], as shown in Figure 1. The BS is located at the top level, the middle level contains all CHs, and other normal sensor nodes stay at the bottom level. Sensing data of sensor nodes is transmitted from the bottom level to the middle level, and finally is forwarded to the top level.

For the development of the following protocol, several assumptions about sensor nodes are listed as follows:

- All sensor nodes are stationary or nearly stationary after they have been deployed into sensing field;
- A unique ID is labeled on each sensor node that can be used to distinguish data sources;
- Each node has limited energy stored in the battery which cannot be recharged, but no energy limitations are inflicted on the BS;
- Each sensor node has the ability of changing its power level dynamically to adapt to different transmission distances.

### 3.2. Radio Model

**Figure 2:**
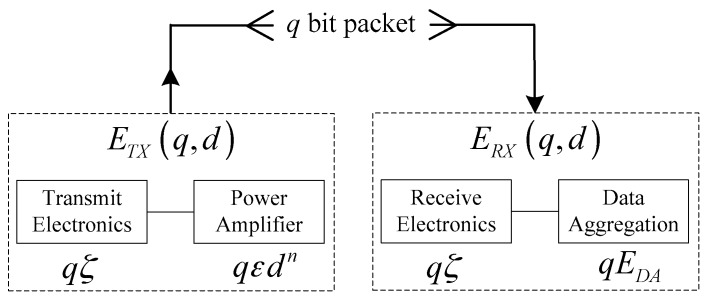
Radio model.

The radio model [30,32,33] adopted in this paper is shown in Figure 2, and it contains a two-ray ground propagation model and free space model according to the distance from data transmitter to receiver.

The energy *E_TX_* consumed by the transmitter to deliver a *q*-bit packet can be calculated by:

(2) $$E_{TX}=\{\begin{array}{cc} \begin{array}{l} q\zeta +q\varepsilon_{f}d^{2}\\ q\zeta +q\varepsilon_{m}d^{4} \end{array} & \begin{array}{l} d<d_{0}\\ d\geq d_{0} \end{array} \end{array}$$

where *ζ* is the energy consumed by the transceiver circuitry for a one-bit packet; *ε_f_* and *ε_m_* are the amplifier characteristic constants with regard to free-space propagation model and two-ray ground reflection model; *d* is the distance from the transmitter to the destination and *d*_0_ is the distance threshold which is computed by:

(3) d0=εfεm

where *ε_f_* and *ε_m_* are the amplifier characteristic constants.

The energy consumed by the receiver to receive a *q*-bit packet can be calculated by:

(4) $$E_{RX}=q\zeta +qE_{DA}$$

where *ζ* is the energy consumed by the transceiver circuitry and *E_DA_* is energy of data aggregating for a one-bit packet.

## 4. Cluster Head Election Game

In this section, we utilize game theory to model the CH election process for clustered WSNs. Our cluster head election game (*CEG*) will be played by sensor nodes within a local area of the network for CH election, and we formally define it as *CEG* = <*N_G_*, *S_G_*, *U_G_*>, where *N_G_* is the set of players, *S_G_* = {*S_i_*} is the set of all feasible strategies and *U_G_* = {*U_i_*} is the set of utility functions. All sensor nodes within a local area act as players and participate in a *CEG*. Each player can rationally and selfishly choose the strategy based on its own interests. For any player *i*, its strategies include “declare myself as CH” (*D*) or “not declare myself as CH” (*ND*). Since energy is a kind of precious resource in WSNs and any node acting as a CH needs to take the responsibility of relaying data for other normal sensor nodes, each sensor node prefers to abdicate the responsibility to other nodes for energy conservation. However, if none of the sensor nodes take the responsibility of being a CH to serve the other nodes, then network failure occurs and no sensor nodes can enjoy the benefit of data transmission. Therefore, each sensor node has to consider the knowledge or expectations of other sensor nodes when making its own decision. We define the utility function *U_i_* of any node *i* when playing the *CEG* as follows:

(5) $$U_{i}=\{\begin{array}{cc} \begin{array}{l} v_{i}-e_{i}\\ v_{i}\\ 0 \end{array} & \begin{array}{l} ifs_{i}=D\;\mathrm{and}\;\forall v_{i},e_{i}\in R,s.t.v_{i}>e_{i}>0\\ ifs_{i}=ND\;\mathrm{and}\;\exists j\in N_{G},s.t.s_{j}=D\\ s_{j}=ND,\;\forall j\in N_{G} \end{array} \end{array}$$

where *v_i_* is the payoff when node *i* chooses the strategy *ND* while there exists at least another node choosing the strategy *D*, and *e_i_* is the extra cost when node *i* chooses *D*.

### 4.1. Achieving the Maximum Payoff

To avoid the case that no sensor nodes choose the strategy *D* when playing the *CEG*, we assume that another chance will be given to all nodes to campaign for the CH when this case occurs for the first time. If still no sensor nodes choose *D*, a penalty mechanism will be introduced to punish the selfish behavior of nodes. We adopt the mixed strategies for all sensor nodes, that is, any node *i* decides whether being the CH based on a probability *p_i_*. Then the payoff *H_i_* for node *i* when playing the *CEG* can be expressed as follows:

(6) $$\begin{array}{c} H_{i}=\left(v_{i}-e_{i}\right)p_{i}+v_{i}\left(1-p_{i}\right)\left(1-\prod_{j\neq i,j\in N_{G}}\left(1-p_{j}\right)\right)+\left(v_{i}-e_{i}\right)p_{i}\prod_{j\in N_{G}}\left(1-p_{j}\right)\\ +v_{i}\left(1-p_{i}\right)\left(1-\prod_{j\neq i,j\in N_{G}}\left(1-p_{j}\right)\right)\prod_{j\in N_{G}}\left(1-p_{j}\right)-b_{i}\prod_{j\in N_{G}}{\left(1-p_{j}\right)}^{2} \end{array}\;\;s.t.\;\left\{ \begin{array}{l} b_{i}\in R,b_{i}>0\\ p_{i}\in R,0\leq p_{i}\leq 1 \end{array}\right.$$

where *v_i_* is the payoff when node *i* chooses the strategy *ND* while there exists at least one CH; *e_i_* is the extra cost when node *i* chooses *D*; *N_G_* is the set of sensor nodes participated in *CEG* and *b_i_* is a positive penalty term inflicted on node *i*.

Equation (6) is a quadratic function depending on probability *p_i_*. Letting *w_i_* be the coefficient of the quadratic term, we can express it as follows:

(7) $$w_{i}=\left(e_{i}-\left(v_{i}+b_{i}\right)\prod_{j\neq i,j\in N_{G}}\left(1-p_{j}\right)\right)\prod_{j\neq i,j\in N_{G}}\left(1-p_{j}\right)$$

where *p_j_* is the probability of being the CH for node *j*.

Letting $\Theta =\prod_{i\in N_{G}}\left(1-p_{i}\right)$, then *w_i_* can be simplified to:

(8) $$w_{i}=\left(e_{i}-\left(v_{i}+b_{i}\right)\frac{\Theta }{1-p_{i}}\right)\frac{\Theta }{1-p_{i}}$$

where *v_i_* is the payoff when node *i* chooses the strategy *ND* while there exists at least one CH; *e_i_* is the extra cost when node *i* chooses *D*; *b_i_* is a positive penalty term inflicted on node *i* and *p_i_* is the probability of being a CH for node *i*. According to the value of coefficient *w_i_*, the payoff function *H_i_* may have two forms that are listed as follows:

- *H_i_* is a concave or linear function when *w_i_* ≥ 0. Under this case, the maximum point of *H_i_* is either *p_i_* = 0 or *p_i_* = 1;
- *H_i_* is a convex function when *w_i_* < 0. Under this case, the maximum point of *H_i_* maybe exists within the open interval (0, 1).

As for the first case, each node decides whether being a CH based on the probability *p_i_* = 0 or *p_i_* = 1 to maximize its own payoff. Which means pure strategy is adopted by sensor nodes under this case. Since we adopt mixed strategies for all nodes as aforementioned, we only consider the second case so that each node decides whether being a CH according to a probability within the range of 0 to 1. Then Θ is positive and we have the following inequality:

(9) $$b_{i}>\frac{1-p_{i}}{\Theta }e_{i}-v_{i}$$

where *b_i_* is a positive penalty term inflicted on node *i*; *p_i_* is the probability of being a CH for node *i*; *v_i_* is the payoff when node *i* chooses the strategy *ND* while there exists at least one CH; *e_i_* is the extra cost when node *i* chooses *D* and Θ is the multiple formative of probabilities not to be the CH for all sensor nodes.

Since *v_i_* > *e_i_* > 0, we find a qualifying value of *b_i_* which can be expressed as follows:

(10) $$b_{i}=\frac{1-p_{i}}{\Theta }e_{i}-e_{i}\frac{E_{i}}{E_{\max}}>0$$

where *E_i_* is residual energy of node *i* and *E*_max_ is the maximum residual energy of sensor nodes participated in the *CEG*.

Combining Equations (6) and (10), the payoff function *H_i_* corresponding to node *i* can be re-expressed as follows:

(11) $$H_{i}=\left(e_{i}\frac{E_{i}}{E_{\max}}-v_{i}\right)\Theta^{2}-e_{i}\Theta +v_{i}-e_{i}p_{i}$$

where *v_i_* is the payoff when node *i* chooses the strategy *ND* while there exists at least one CH; *e_i_* is the extra cost when node *i* chooses *D*; Θ is the multiple formative of probabilities not to be the CH for all sensor nodes and *p_i_* is the probability of being a CH for node *i*.

We calculate the derivative of *H_i_* with regards to *p_i_* as follows:

(12) $$\frac{\partial H_{i}}{\partial p_{i}}=-2\left(e_{i}\frac{E_{i}}{E_{\max}}-v_{i}\right)\frac{\Theta^{2}}{1-p_{i}}+e_{i}\frac{\Theta }{1-p_{i}}-e_{i}$$

Letting this derivation to be zero, we can get the maximum point *p_i_*′ of *H_i_* as follows:

(13) $$p_{i}{}^{\prime }=1-\Theta^{\prime }-2\left(\frac{v_{i}}{e_{i}}-\frac{E_{i}}{E_{\max}}\right)\Theta^{\prime }{}^{2}<1,\;s.t.\;p_{i}{}^{\prime }\in R,0\leq p_{i}{}^{\prime }\leq 1$$

where *v_i_* is the payoff when node *i* chooses the strategy *ND* while there exists at least one CH; *e_i_* is the extra cost when node *i* chooses *D*; *E_i_* is residual energy of node *i*; *E*_max_ is the maximum residual energy of sensor nodes participated in the clustering game and Θ’ is the multiple formative $\left(1-p_{i}{}^{\prime }\right)\prod_{j\in N_{G},j\neq i}\left(1-p_{j}\right)$.

Equation (13) shows that the maximum point of *H_i_* not only depends on the strategy of node *i*, but also relies on the combination of all other sensor nodes that participated in the *CEG*. A sensor node which has more energy has a bigger probability to be CH at the maximum point. In addition, a sensor node with too little energy may have a negative value at the maximum point that is inconsistent with the term probability. As *H_i_* is a convex function, its reasonable maximum point within the range (0, 1) can be expressed as follows:

(14) $$p_{i}{}^{m}=\max\left\{0,p_{i}{}^{\prime }\right\},\;s.t.\;i\in N_{G},p_{i}{}^{m}\in R,0\leq p_{i}{}^{m}\leq 1$$

where *p_i_*′ is the previous optimal probability of being a CH for node *i* that can maximize its own payoff *H_i_* and *p_i_^m^* is the revised optimal probability of being a CH for node *i*.

Equation (14) indicates that the payoff of node *i* can be maximized when its probability to be CH is either 0 or *p_i_*′.

### 4.2. Searching for the Equilibrium Strategy

In this subsection, we search for the equilibrium strategy under which no sensor node has incentives to enhance its own payoff by unilaterally changing the selected strategy. Letting *P* be the strategy combination of all sensor nodes in set *N_G_*, *P*_−*i*_ be the strategy combination of sensor nodes excepting node *i* and *N* be the total number of sensor nodes. Then we define the NE strategy of the *CEG* as follows:

**Definition 1.**  *A strategy combination P = {p_1_, p_2_, …, p_N_} is an NE point of the CEG, when inequality H_i_(P) ≥ H_i_(P_−i_, p_i_) is satisfied for any sensor node i in set N_G_ [28]*.

**Proposition 1.**  *The strategy combination P^NE^ as shown in Equation (15) is a NE point of the CEG*.

(15) $$P^{NE}=\left\{p_{1}{}^{m},p_{2}{}^{m},\cdots ,p_{i}{}^{m},\cdots ,p_{N}{}^{m}\right\},\;s.t.\;P^{NE}\in R^{N}\mathrm{and}\forall i\in N_{G},0\leq p_{i}{}^{m}\leq 1$$

*where p_i_^m^ is the probability of being the CH for sensor node i which is calculated by Equation (14), and N is the total number of nodes participated in the CEG*.

**Proof.**  Since each sensor node is modeled as a rational and selfish individual, it expects to abdicate the duty of serving as a CH to other nodes for energy saving. However, each node also is afraid that no profits can be acquired from data transmission, as it cannot know other nodes’ decisions in advance. Then any node *i* decides whether being the CH according to a probability *p_i_*, we recalculate the payoff function *H_i_*(*P*_−*i*_, *p_i_*) for node *i* as follows:

(16) $$H_{i}\left(P_{-i},p_{i}\right)=\left(e_{i}\frac{E_{i}}{E_{\max}}-v_{i}\right)\Theta_{-i}{}^{2}{\left(1-p_{i}\right)}^{2}-e_{i}\Theta_{-i}\left(1-p_{i}\right)+v_{i}-e_{i}p_{i}$$

where *v_i_* is the payoff when node *i* chooses the strategy *ND* while there exists at least one CH; *e_i_* is the extra cost when node *i* chooses *D*; *E_i_* is residual energy of node *i*; *E*_max_ is the maximum residual energy of sensor nodes participated in the *CEG*; Θ_−*i*_ is the multiple formative of probabilities not to be the CH for all sensor nodes excepting node *i* and *P*_−*i*_ is the strategy combination excepting node *i*. □

Since *v_i_* > *e_i_* > 0, *E_max_* ≥ *E_i_* > 0 and Θ_−*i*_ > 0, then *H_i_*(*P*_−*i*_, *p_i_*) is a quadratic convex function depending on probability *p_i_*. And the maximum point of function *H_i_*(*P*_−*i*_, *p_i_*) is the probability *p_i_*′ calculated by Equation (13). If *p_i_*′ < 0, then *H_i_*(*P*_−*i*_, *p_i_*) is monotonically decreasing with the increase of *p_i_* from 0 to 1. That is:

(17) $$\forall p_{i}\in [0,1]s.t.H_{i}\left(P_{-i},p_{i}\right)\leq H_{i}\left(P_{-i},0\right).$$

If *p_i_*′ ≥ 0, then *H_i_*(*P*_−*i*_, *p_i_*) firstly increases with the increase of *p_i_* from 0 to *p_i_*′, and then decreases with the increase of *p_i_* from *p_i_*′ to 1. Hence, we have:

(18) $$\forall p_{i}\in [0,1]s.t.H_{i}\left(P_{-i},p_{i}\right)\leq H_{i}\left(P_{-i},p_{i}{}^{\prime }\right).$$

Based on Equations (14), (17) and (18), we have:

(19) $$\forall i\in N_{G},\forall p_{i}\in [0,1]s.t.H_{i}\left(P_{-i},p_{i}\right)\leq H_{i}\left(P_{-i},p_{i}{}^{m}\right).$$

which means that any sensor node *i* in set *N_G_* can maximize its own payoff *H_i_*(*P*_−*i*_, *p_i_*) when it decides whether being the CH according to the probability *p_i_^m^* calculated by Equation (14).

As the selfish behaviors of sensor nodes, each node in set *N_G_* will decide whether being the CH according to the probability calculated by Equation (14) simultaneously for maximizing its payoff. Then the selected strategy combination is *P^NE^* that calculated by Equation (15). Any sensor node *i* in set *N_G_* has no incentive to deviate from it unilaterally, because the inequality *H_i_*(*P*_−*i*_*^NE^*, *p_i_^m^*) ≥ *H_i_*(*P*_−*i*_*^NE^*, *p_i_*) is always true according to Equations (14)–(19). Here *P*_-*i*_*^NE^* is the strategy combination of sensor nodes in set *N_G_* excepting node *i*. Hence, the strategy combination *P^NE^* is a NE point of the *CEG* according to Definition 1.

**Proposition 2.**  *The strategy combination P^Z^ = {0, 0,…, 0} is not a NE point of the CEG*.

**Proof.**  Letting *P*_−*i*_*^Z^* be the strategy combination of sensor nodes in set *N_G_* excepting node *i*, then the payoff of node *i* for the strategy combination (*P*_−*i*_*^Z^*, *p_i_*) can be calculated by:

(20) $$H_{i}\left(P_{-i}{}^{Z},p_{i}\right)=-\left(v_{i}-e_{i}\frac{E_{i}}{E_{\max}}\right){\left(1-p_{i}\right)}^{2}+v_{i}-e_{i}$$

where *v_i_* is the payoff when node *i* chooses the strategy *ND* while there exists at least one CH; *e_i_* is the extra cost when node *i* chooses *D*; *E_i_* is residual energy of node *i*; *E*_max_ is the maximum residual energy of sensor nodes participated in the *CEG* and *p_i_* is the probability of being a CH for node *i*. □

Equation (20) shows that node *i* has incentives to enhance its own payoff by deviating from the strategy *p_i_* = 0, that is *P^Z^* is not a NE point according to Definition 1.

Based on the above two propositions, we can conclude that there is at least one node that has a positive probability to be CH at the NE point *P^NE^* which is given by Equation (15). In addition, the value of Θ at this NE point is within the range (0, 1) according to Equations (13) and (14). However, how to calculate the probability of being a CH for each sensor node at the NE point of *CEG* still presents great challenges. We can first calculate the value of Θ at the NE point, expressed as Θ*^NE^*. And then we can calculate the probability to be CH at the NE point for each node based on Equations (13) and (14). For the purpose of searching the root of Θ*^NE^*, we can employ a binary search algorithm or Newton’s method to solve the following optimization problem:

(21) $$\min f\left(\Theta \right)=\left|\Theta -\prod_{i\in N_{G}}\left(1-p_{i}^{m}\left(\Theta \right)\right)\right|,\;s.t.\Theta \in R,0<\Theta <1$$

where *p_i_^m^* is the optimal probability of being a CH for node *i* that can maximize its own payoff; Θ is the multiple formative of probabilities not to be the CH for all sensor nodes and *N_G_* is the set of sensor nodes that participated in the *CEG*.

Since the binary search method has lower complexity and higher convergence speed than Newton’s method, it is more suitable for sensor nodes which have limited computation abilities. The details of the binary search method are as follows:

- First step: Input the minimum value of Θ (we denote it as *min*), the maximum value of Θ (we denote it as *max*) and error precision *err*;
- Second step: Calculate the expression (*min* + *max*)/2, and assign the result to Θ;
- Third step: If |*max* − *min*| < *err*, or *f’*(Θ) < *err*, terminate the iteration process and output the final result of Θ, which is denoted as Θ*^NE^*; Otherwise, go to the next step;
- Forth step: If *f’*(Θ)*f’*(*min*) > 0, assign the value of Θ to *min*; Otherwise, assign the value of Θ to *max*;
- Last step: Go to the second step to continue the iteration process until the maximal number of iterations is completed.

During the above iteration process, the initial value of *min* is set to be 0.001, the initial value of *max* is 0.999, the value of *err* is 0.005 and the maximal number of iteration is 20.

Finally, we give an example of the NE solution *P^NE^* for our *CEG* as shown in Table 1. Here we only list the result for a *CEG* which contains in total 8 sensor nodes. These nodes are distributed in the same local area of the network and act as players to join in the same *CEG*. Moreover, they hold different values about the distance to BS, number of neighbor nodes and residual energy.

## 5. The Protocol Details 

**Figure 3:**
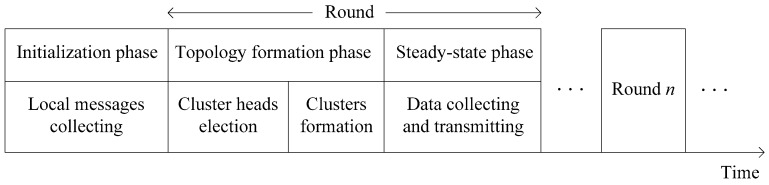
Procedure of the proposed game theoretic clustering protocol.

In this section, the details of our protocol will be described and we call it a game theoretic approach for balancing energy consumption in clustered WSNs (GTAB). The procedure [30,32] of GTAB is composed of a one-time initialization phase and lots of repeated rounds which can further be divided into topology formation and steady-state phases, as shown in Figure 3.

In initialization phase, a “Start” message will be broadcasted by BS within the whole network, so that the distances to BS can be estimated by sensor nodes in the network by utilizing received signal strengths. Then, each node broadcasts a “Hello” message which contains its node ID within the communication radius *R*. Each node can know all its neighbor nodes as it will receive other nodes’ messages. Followed the initialization phase is topology formation phase, where CHs will be selected firstly. Then, an appropriate CH will be chosen by each node for cluster joining. Based on this clustered network topology, each node begins to collect data from the surrounding environment, and transmits the data to BS at steady-state phase. To avoid the communication interference during wireless communication, each CH will create an individual Time Division Multiple Access (TDMA) schedule for all its member nodes. Then a sensor node goes dormant during most of the time. Only when its own time slot arrives, it will be awakened for data transmission. After a CH has received all member nodes’ data, it will transmit the data to BS according to a randomly selected Code Division Multiple Access (CDMA) code. In the following of this section, we will emphatically introduce the topology formation phase that can be divided into CHs election and clusters formation phases.

### 5.1. CHs Election

To select the appropriate CHs in the network, each node holds a probability to be CH, and decides whether being a CH based on this probability. In the first round of CHs election, each node decides whether being a CH according to the expected probability which is expressed as follows:

(22) $$p_{ept}=\frac{S}{\pi {\left(\varepsilon R\right)}^{2}n}$$

where *S* is sensor field size; *R* is communication radius of each node; *n* is the total number of sensor nodes in the network and *ε* is the radius adjustment factor within the range of (0, 1).

We provide some explanations about the significance of the expected probability: as the communication radius of each node is *R*, the area covered by a CH is exactly equal to *πR*^2^. Then the expected number of CHs in the network is S/(*πR*^2^). However, CHs are randomly selected based on this expected probability, so we need to select more CHs for further reselection and the radius adjustment factor *ε* is introduced to update the expected number of CHs. Finally, the expected probability of being the CH can be expressed by Equation (22).

For a sensor node, if it is finally selected as the CH, then it will take the responsibilities of receiving, aggregating and forwarding data for its member nodes after the cluster formation phase is finished. Most importantly in our protocol, it will calculate the probabilities to be CH in the next round for its member nodes.

For any CH node *ch_k_*, it checks the number *n_k_* of member nodes that are still alive after the cluster topology is constructed. If *n_k_* < 1, *ch_k_* still has to campaign for the CH in the next round according to the expected probability, since there is no other CH candidate within the cluster. If *n_k_* = 1, the probability to be CH of *ch_k_* in the next round is set to be zero to avoid continuous working as CH, and the only CM node will campaign for the CH in the next round according to the expected probability. If *n_k_* > 1, there are more than one CH candidate within the cluster, then the probability to be CH for *ch_k_* in the next round is also set to be zero. Moreover, a *CEG* and probability transformation mechanism will be introduced by *ch_k_* for the purpose of calculating the probabilities to be CH for its member nodes.

As for the third case, all active member nodes of *ch_k_* will act as players to join a *CEG*. By solving the Optimization Problem (21), *ch_k_* can calculate the absolute equilibrium probabilities to be CH in the next round for its member nodes according to Equations (13)–(15). However, several parameters need to be determined in *CEG*. Firstly, the number of players that participate in the *CEG* is exactly equal to the number *n_k_* of active member nodes of *ch_k_*. Secondly, the residual energy of each player can be acquired by *ch_k_* during the process of clusters formation phase. At last, to determine *v_i_* and *e_i_* for player *i*, we define the payoff of a player as the number of data bits per unit of energy consumed to transmit the data to CH or BS [30]. Then when player *i* chooses the strategy *ND* while there exists at least another player choosing the strategy *D*, its payoff *v_i_* can be calculated by:

(23) $$v_{i}=q/Ecm_{i}$$

where *q* is the length of data packet, and *Ecm_i_* is the amount of energy consumed to transmit the data packet to the corresponding CH that can be expressed as follows:

(24) $$Ecm_{i}=q\zeta +q\varepsilon_{f}{\left(\frac{2}{3}R\right)}^{2}$$

where *ζ* is the energy consumed by the transmitting or receiving circuitry; *ε_f_* is the amplifier characteristic constant corresponding to free-space propagation model; *R* is the communication radius of each node, and the average distance between a member node and its corresponding CH is 2*R*/3.

When player *i* chooses *D* as its strategy, the extra cost *e_i_* can be calculated by:

(25) $$e_{i}=v_{i}-\frac{q}{Ech_{i}}$$

where *v_i_* is the payoff when node *i* chooses the strategy *ND* while there exists at least one CH; *Ech_i_* is the amount of energy consumed by player *i* to transmit the packet with length *q* to BS when it acts as the CH, and *Ech_i_* can be expressed as follows:

(26) $$Ech_{i}=q\zeta Nb_{i}+qE_{DA}\left(Nb_{i}+1\right)+q\varepsilon_{m}d_{i}^{4}$$

where *q* is data packet length; *ζ* is the energy consumed by the transceiver circuitry; *Nb_i_* is the number of neighbor nodes of player *i*; *E_DA_* is the energy consumed for aggregating a one-bit data packet; *ε_m_* is the amplifier characteristic constant regard to two-ray ground reflection model and *d_i_* is the distance between player *i* and BS.

After the *CEG* is finished, *ch_k_* can get the absolute equilibrium probability *p_i_^m^* to be the CH in the next round for any member node *i*. In fact, sensor nodes usually are unevenly distributed in the sensing field which results in uneven clustering. Some clusters may have more member nodes joining the *CEG* than other clusters so that the nodes in different clusters may have different levels of absolute equilibrium probability. To avoid this case, a probability transformation mechanism is adopted to transform absolute equilibrium probability into relative equilibrium probability. For any member node *i*, its relative equilibrium probability *p_i_^r^* to be CH in the next round can be expressed as follows:

(27) $$p_{i}{}^{r}=p_{i}{}^{m}-\sum_{j=1}^{n_{k}}p_{j}{}^{m}/n_{k}+p_{ept}$$

where *p_i_^m^* is the absolute equilibrium probability of being a CH for node *i*; *n_k_* is the number of active member nodes in the cluster to which node *i* belongs and *p_ept_* is the expected probability calculated by Equation (22). After this transformation is finished, sensor nodes in different clusters have the same average probability to be CH.

Since each sensor node decides whether to be a CH based on its corresponding probability to be a CH, this random CHs selection mechanism may cause an uneven distribution of clusters. To avoid this case, a sensor node which successfully self-selected as the CH acts as CH tentatively and will further run for the final CH role. A tentative CH will firstly broadcast a tentative CH election message (including node ID, node status and residual energy) within communication radius *R*. Then it can know the neighbor heads by receiving this kind of messages from other heads. A tentative CH which holds more energy than its neighbors will be the final CH, and an election message including node ID and node status will be broadcasted within radius *R*. A tentative CH which has received the final CH election message will broadcast a quitting CH election message to abdicate the chance to its neighbor heads.

### 5.2. Clusters Formation

After all CHs have been determined, normal nodes will select the appropriate CHs to join clusters. For a normal sensor node which has received one or more final CH election messages, it first estimates the distances to these CHs by utilizing received signal strengths. Then, a joining message including node ID, CH ID and residual energy will be sent to the nearest CH to join a cluster. If a normal node has not received any final CH election messages, then it is not covered by any CH and becomes an isolated node. Each isolated sensor node has to change its power level to enable its communication with the nearest CH, and then joins the corresponding cluster. Once a CH node has received all joining messages, it first calculates the probabilities to be CH for these normal nodes which have sent the joining messages. Then it replies acceptance messages to these normal nodes including node ID, and the probability to be CH in the next round.

## 6. Performance Evaluations

In this section, the performance of our proposed protocol GTAB will be evaluated via simulations executed by using MATLAB. Since LEACH is one of the most famous cluster-based routing protocols, and CROSS and LGCA are game theoretical cluster-based routing protocols, we will evaluate the performance of GTAB by comparing it with these three protocols. The parameters that adopted in our simulations are given in Table 2.

Besides, the expected proportion of CHs is set to be 0.05 for LEACH. Since the predefined parameter *w* has important influence on the performance of the network in CROSS and LGCA, we find its optimal value 0.02 that can maximize the network lifetime through plenty of experiments. And we set *w* to be 0.02 in our following simulations.

To evaluate the performance of GTAB comprehensively, we will conduct the simulations under various network conditions. Firstly, different network sizes are considered during the simulation to find the effect of network scale on the network lifetime, round until the last node dies, average number of packets per node and average number of CHs per round; then, different communication radiuses of sensor nodes are considered in the simulation to find the influence of communication radius on network lifetime; At last, to find the effect of node density on the network lifetime, the simulation is performed for the networks with different node densities. In the rest of this section, we begin our simulations under the case that network size ranges from 100 × 100 to 200 × 200 m^2^, node density is 1 node/100 m^2^ and communication radius is 35 m.

**Figure 4:**
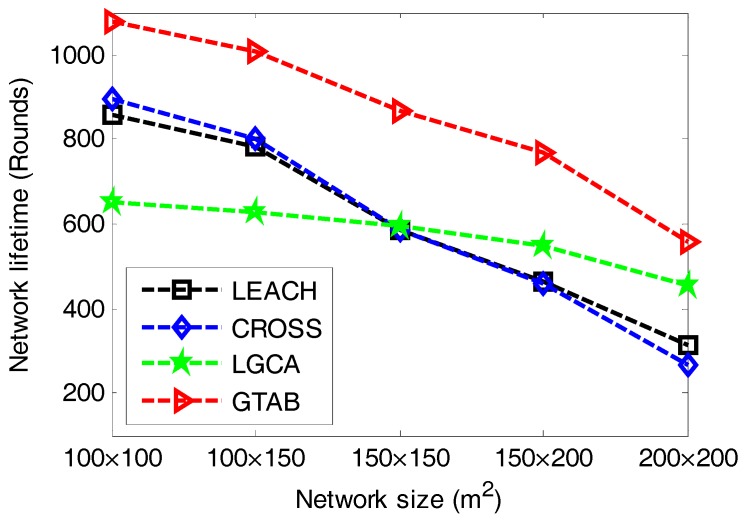
Network lifetime versus different network sizes.

The most important criterion for performance evaluation in clustering is network lifetime, which is defined as the lifespan of the node which first exhausts its energy in the network. Figure 4 gives the comparison of network lifetime versus different network sizes among LEACH, CROSS, LGCA and GTAB. From Figure 4, we can see that our proposed protocol GTAB outperforms other three protocols regarding the network lifetime for all network size cases. This is because GTAB has integrated with our proposed clustering model and game theoretical model. Specifically, by playing our clustering game each node not only acquires the equilibrium between energy cost and benefit from data transmission, but also achieves its own maximum payoff. To analyze the degree of improvement to the network lifetime for our proposed clustering model and game theoretical model, Table 3 lists the network lifetimes of LEACH, routing scheme which only using our proposed clustering model (Only-clustering) and GTAB under the case that the network size is 100 × 100 m^2^. From Table 3 we can find that compared with LEACH, the routing scheme that only utilizing our proposed clustering model has improved the network lifetime by 15%. After the game theory is introduced into our clustering model, the network lifetime has been improved by 26%.

In addition, Figure 4 also shows that all the four protocols have reduced network lifetimes when the network size increases. This is because the average distance between CHs and BS increases with the increase of network size. We can find that the network lifetime in LGCA is shorter than that in CROSS when the network size is smaller than 150 × 150 m^2^, but the opposite is true when the network size is larger than 150 × 150 m^2^. This condition indicates that the LGCA protocol is more suitable for use in larger scale networks than CROSS since its implementation is completely distributed.

**Figure 5:**
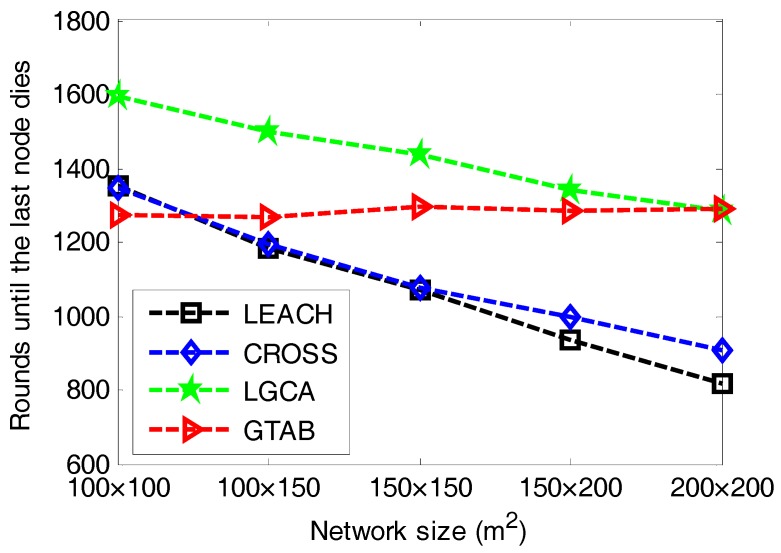
Rounds until the last node dies versus different network sizes.

The round until the last node dies is also an important criterion for performance evaluation in clustering. Figure 5 gives the comparison of the rounds until the last node dies versus different network sizes among LEACH, CROSS, LGCA and GTAB. Figure 5 shows that when the network size increases, LEACH, CROSS and LGCA have downward trends regarding the round until the last node dies, but our proposed protocol GTAB has a relative stable level about the round until the last node dies. This case indicates that with the increase of the network size, the duration of the network decreases when adopting the protocol LEACH, CROSS or LGCA, but the effect of network size on the network duration is relatively smaller when adopting the GTAB scheme. As mentioned before, the network lifetime when using GTAB decreases with the increase of network size, which results in the increase of round interval from the first node dies to the last node dies. This means that GTAB runs more energy-efficiently when network size increases so that it is very suitable for large scale networks.

**Figure 6:**
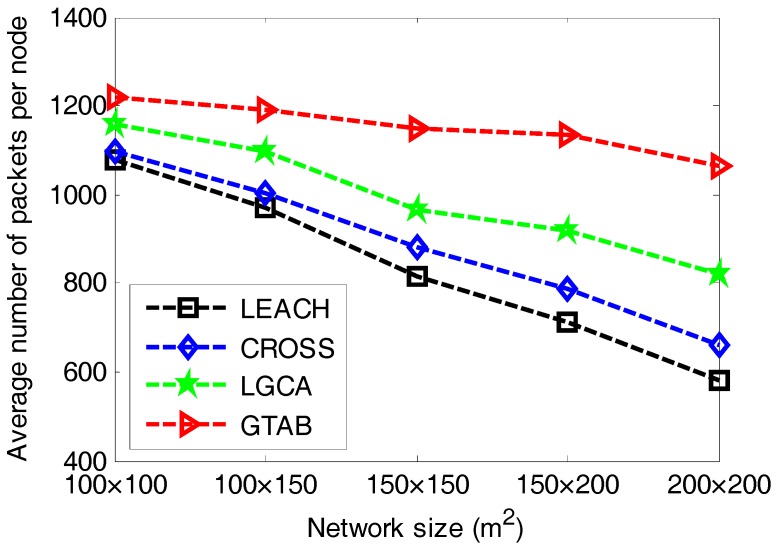
Average number of packets per node versus different network sizes.

Figure 6 gives the comparison of average number of packets per node versus different network sizes among LEACH, CROSS, LGCA and GTAB. Figure 6 shows that GTAB has the greatest average number of packets per node among these protocols for all cases of network size, which means the energy efficiency of GTAB is the highest. In addition, the difference of average number of packets per node between GTAB and other three protocols increases with the increase of network size. This phenomenon implies that compared with other three protocols, GTAB has a relatively increasing energy efficiency when the network size increases.

**Figure 7:**
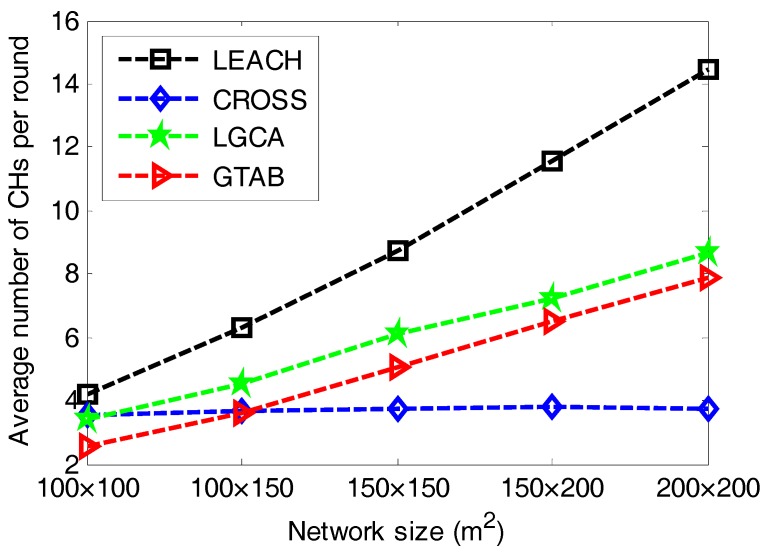
Average number of CHs per round versus different network sizes.

Figure 7 gives the comparison of average number of CHs per round versus different network sizes among LEACH, CROSS, LGCA and GTAB. From Figure 7, we can see that LEACH has the most number of CHs per round for all cases of network size. It also has the highest increase rate of the number of CHs per round when the network size increases. This is due to the constant expected percentage of CHs in LEACH. The number of CHs in CROSS is relatively stable. This is because for any sensor node in CROSS, once it has been selected as a CH, then it has no chance to be the CH again until all other sensor nodes have served as CHs in the network. Figure 7 also shows that with the increase of network size, the average number of CHs per round increases in both LGCA and GTAB. Furthermore, LGCA has more average number of CHs per round than GTAB for all cases of network size. This can be explained as follows: due to the completely distributed implementation of both LGCA and GTAB, CHs will be selected within every localized area. Thus, the larger the network size becomes, the more the number of CHs that will be selected. Moreover, the probability of being the CH in LGCA strongly depends on the predefined parameter *w*, and it increases with the decrease of *w*.

Then in the following simulations, the network size is set to be 100 × 100 m^2^ and the node density is 1 node/100 m^2^. We first show the detail of number of nodes that still alive in each round when communication radius is 35 m. Then we gives comparison of network lifetime versus different communication radiuses among these protocols, for the purpose of analyzing the effect of the communication radius on network lifetime.

**Figure 8:**
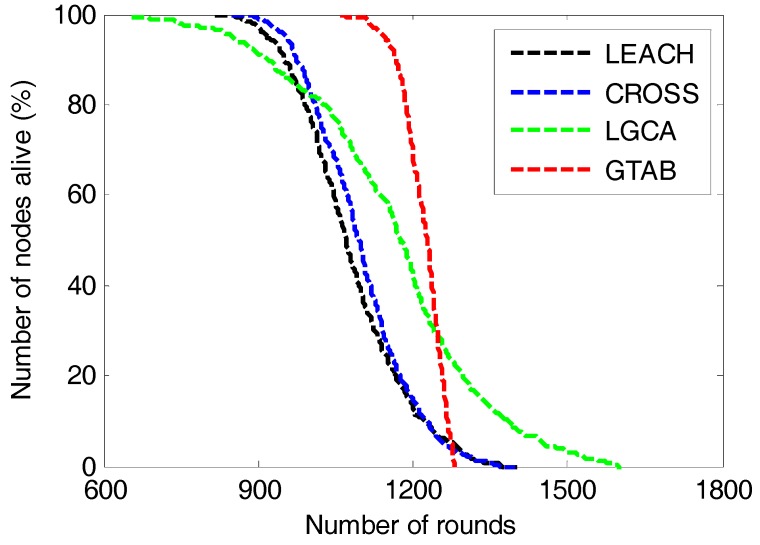
Number of nodes alive versus the number of rounds.

Figure 8 gives the comparison of number of nodes alive versus the number of rounds among LEACH, CROSS, LGCA and GTAB. The round interval from the first node dies to the last node dies in our proposed protocol GTAB is the shortest among these protocols, and that in LGCA is the longest. This implies that the energy balancing performance of GTAB is the best, and that of LGCA is the worst. This is because a penalty mechanism is introduced in GTAB to punish the selfish sensor nodes which abandon the duty of serving as CHs for energy saving, so that sensor nodes which have more energy have bigger probabilities to be CHs that can well balance the energy among nodes. However in LGCA, once a sensor node has served as a CH, it will not be selected as the CH again until all its neighbor nodes have served as the CHs. Thus sensor nodes with more neighbor nodes have less chance to be CHs which results in a poor energy balancing performance among nodes. Figure 8 also shows that LEACH and CROSS almost have equivalent round intervals from the first node dies to the last node dies, which means these two protocols have equivalent energy balancing performance among sensor nodes. This phenomenon can be explained as follows: as the average number of CHs per round in CROSS and LGCA is approximately equal to 4 when the network size is 100 × 100 m^2^. For a sensor node in CROSS, once it has been selected as the CH, it will not be the CH again until all other nodes have served as the CHs in the network. Then once a node has been the CH in one round, it will have no chance again to be the CH in the following 24 rounds. In other words, a sensor node will be selected as the CH once every 25 rounds in CROSS, and this mechanism of sensor nodes taking turns to be CHs is very similar to that used in LEACH.

**Figure 9:**
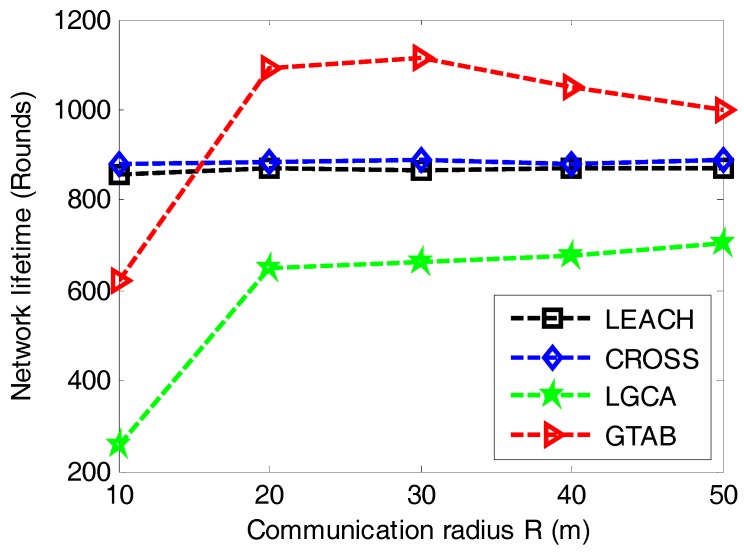
Network lifetime versus different communication radiuses of sensor nodes.

In order to analyze the trend of network lifetime when communication radius of sensor nodes increases, Figure 9 gives the comparison of network lifetime versus different communication radiuses of sensor nodes among LEACH, CROSS, LGCA and GTAB. From Figure 9, we can find that there is no effect of the communication radius on network lifetime in LEACH and CROSS. This is because these two protocols have no limitations to sensor nodes with regard to the communication radius. Both GTAB and LGCA display their shortest network lifetimes when the communication radius equals the minimum value of 10 m, this is because too many CHs are selected in both GTAB and LGCA when the communication radius is very small. With the increase of communication radius, the network lifetime in GTAB first increases and then decreases gradually, and that in LGCA keeps increasing slowly. We can explain this phenomenon as follows: too many CHs will be selected if the communication radius is very small in GTAB and LGCA. Less CHs will be selected when the communication radius increases which results in a heavier data burden per CH. Thus the network lifetime will be affected negatively if the communication radius keeps increasing or decreasing. However, we only find the downtrend of network lifetime in GTAB but not in LGCA when communication radius increases, this case ascribes to that GTAB achieves the optimal number of CHs earlier than LGCA with the increase of communication radius.

**Figure 10:**
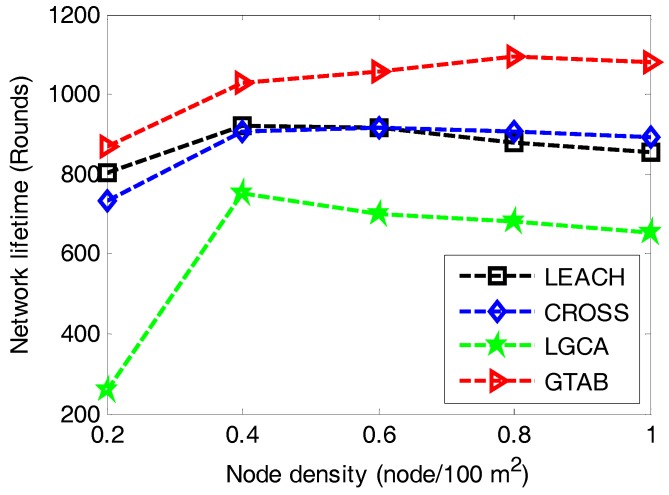
Network lifetime versus different node densities.

At last, to find the effect of node density on network lifetime, we conduct a simulation under the case that network size is set to be 100 × 100 m^2^, communication radius is 35 m and node density increases from 0.2 to 1 node/100 m^2^. Figure 10 gives the comparison of network lifetime versus different node densities among LEACH, CROSS, LGCA and GTAB. It shows that our proposed protocol GTAB outperforms other three protocols for all cases of the node density. All four protocols display their shortest network lifetimes simultaneously when the node density is the minimum value 0.2. This is because sensor nodes become CHs more frequently for a network with lower node density. Moreover, with the increase of node density, the network lifetimes of the four protocols first increase and then decrease gradually. This is because the average data burden per CH becomes heavier for a network with bigger node density.

## 7. Conclusions and Future Work

In this paper, we present a game theoretic approach for balancing energy consumption in clustered WSNs, which is called GTAB. The CH election process is modeled as a clustering game, and sensor nodes in a close proximity are considered as players which will participate in the game for CH election. We construct a novel convex payoff function for each sensor node in the game, where a penalty mechanism is introduced to well capture this node’s practical behaviors. Then the sensor nodes which hold more energy are compelled to compete for the CH more passionately. Through convex optimization, we get the NE strategy of this clustering game. Each sensor node decides whether to act as the CH based on this NE strategy for a tradeoff between providing required services and energy saving. Specifically, its own payoff can be maximized. Extensive simulation results have verified that our protocol can effectively improve the performance of energy balancing among nodes, and the network lifetime is significantly enhanced.

As future work, we will continue to use game theory to study clustering in WSNs from three aspects: Firstly, we will adopt game theory to solve the cluster area allocation problem in multi-hop clustered WSNs, where CHs in the network cooperatively transmit data to BS by a multi-hop communication method so that unequal clustering is required to avoid energy hole problem; Secondly, we will use game theory to solve the CH election problem in energy harvesting WSNs, where sensor nodes are equipped with energy harvesting devices that can harvest energy from the ambient environment; At last, we will utilize game theory to address the security issue in cluster-based WSNs, and both authentication and trust based mechanisms will be studied.

## Figures and Tables

**Table 1:** An example of the NE solution for our *CEG*.

Nodes Participated in the *CEG*	Distances to BS (m)	Number of Neighbors	Residual Energy (J)	NE Solution (*P^NE^*)
Node 1	78.8692	9	0.2934	0
Node 2	98.5443	4	0.2781	0
Node 3	93.2947	1	0.3720	0
Node 4	86.7700	5	0.4303	0.0522
Node 5	96.0795	5	0.3040	0
Node 6	91.2931	7	0.3965	0.0319
Node 7	74.0076	14	0.4711	0.2370
Node 8	87.9603	7	0.2531	0

**Table 2:** Parameter setup.

Parameters	Values
Basic energy *E_b_* (J)	0.5
Random energy exponent *α*	0.1
Packet size *q* (bits)	3000
Control packet size (bits)	300
*ζ* (nJ/bit)	50
*ε_m_* (pJ/bit/m^4^)	0.0013
*ε_f_* (pJ/bit/m^2^)	10
*E_DA_* (nJ/bit/message)	5
Radius adjustment factor *ε*	0.8

**Table 3:** Lifetimes of the network when using LEACH, Only-clustering and GTAB.

Routing Scheme	Network Lifetime
LEACH	856
Only-clustering	986
GTAB	1081
